# Practical Therapeutics, Etc.

**Published:** 1871-10

**Authors:** 


Practical Therapeutics, considered chiefly with reference to Articles of the Materia Medica. By Edward John Waring, M. D., F.L.S. Second American, from the third London edition. Lindsay & Blakiston, Publishers. Price, cloth, $5.00; sheep, $6.00.

This valuable work has been thoroughly revised, and to a great extent re-written. The many new and important therapeutical facts and discoveries which have been developed within the last few years, necessitated the addition of a considerable amount of new matter. By a careful abridgement and consideration of certain portions of the work, however, space has been made for these additions, without any increase in the size of the volume.

Among the new articles considered are chloral, bromide of mercury, iodide of methyl, protoxide of nitrogen, sandelwood oil, etc.

Extended notices are also given of such articles as bromide potassium, Calabar bean, carbolic and sulphurous acids, permanganate of potash, and the alkaline hypophosphites and hyposulphites, which, although not strictly new, have only of late years had their claims, as valuable and important therapeutical agents, fully recognized.

